# Evaluation of Heart Rate Variation Analysis during Rest and Tilting in Patients with Temporal Lobe Epilepsy

**DOI:** 10.1155/2011/829365

**Published:** 2011-07-14

**Authors:** Hanna Ansakorpi, Juha T. Korpelainen, Kalervo Suominen, Uolevi Tolonen, Risto Bloigu, Vilho V. Myllylä, Jouko I. T. Isojärvi

**Affiliations:** ^1^Department of Neurology, University of Oulu, P.O. Box 5000, 90014 Oulu, Finland; ^2^Department of Clinical Neurophysiology, University of Oulu, P.O. Box 5000, 90014 Oulu, Finland; ^3^Medical Informatics Group, University of Oulu, P.O. Box 5000, 90014 Oulu, Finland; ^4^UCB, Inc., 8010 Arco Corporate Drive, Raleigh, NC 27617, USA

## Abstract

*Objective*. To evaluate spectral heart rate (HR) variation using short-term ECG recordings at rest and during the tilt table test. *Methods*. The values of spectral components of total power (TP), high-frequency power (HF), low-frequency power (LF) and LF: HF ratio were measured at rest and during the head-up tilt in patients with temporal lobe epilepsy (TLE) and their control subjects. *Results*. Compared to the control subjects, patients with TLE had lower HF (*P* < 0.05) and LF : HF ratio (*P* < 0.05) at rest and lower TP (*P* < 0.001), HF (*P* < 0.05), and LF (*P* < 0.05) during the head-up tilt. Upon changing from supine to standing position TP (*P* < 0.05) and LF (*P* < 0.05) were attenuated in patients with TLE compared to the control subjects. *Conclusion*. These results suggest that spectral analysis of HR variation from ECG recordings of short duration may add value to assessment of autonomic nervous system function using autonomic cardiac tests in patients with TLE.

## 1. Introduction

Epilepsy is well known to be associated with various autonomic phenomena [1, 2]. Both ictal and interictal changes in cardioregulatory function of the autonomic nervous system (ANS) have been described in previous studies [3–19]. Although their prognostic role is not yet established in patients with epilepsy, similar changes in heart rate (HR) variation described in patients with epilepsy have been detected in other chronic diseases and shown to be associated with an unfavourable prognosis in patients with various heart conditions and cerebrovascular diseases [20–25]. Moreover, it has been suggested that unstable cardiac and respiratory control of the ANS may contribute to sudden unexpected death in epilepsy (SUDEP) [26–33]. 

Autonomic cardiovascular regulation can be assessed with different methods [28, 34]. The test pattern of autonomic cardiac function is performed under standardised environmental conditions. The adaptive responses of the ANS to various stimuli are assessed during the test [34]. Although the analysis of HR variation from an ECG of short duration obtained during the autonomic cardiac function tests may not be as sensitive as spectral analyses of HR variation from 24-hour Holter recordings in revealing subtle abnormalities in linear as well as dynamic fluctuations of HR variation, the method provides valuable information when conducted according to the strict protocol developed [34, 35]. Spectral analyses of HR variation can also be performed from the short ECG recordings obtained during the autonomic function tests. This allows assessment of the ability of the autonomic cardioregulatory functions to maintain the homeostasis of the body in standardized dynamic conditions. 

In this study, spectral measures of HR variation were analysed post hoc from ECG data obtained during the normal breathing and tilt-table tests of the autonomic cardiac reactivity evaluation in patients with temporal lobe epilepsy (TLE) and control subjects. The results of the autonomic cardiac function tests of these patients have been previously published [8]. Spectral analysis of HR variation from 24-hour ECG recordings has also been conducted in these same patients, and the results showed decreased overall autonomic cardiac regulation associated with TLE [10]. A recent study by DeGiorgio et al. showed that low values of certain parameters of HR variability correlate with clinical risk markers of SUDEP [36]. Although the risk evaluation of SUDEP cannot be solely based on ANS function, it is important to try to develop easily accessible methods to detect autonomic instability in epilepsy patients. Our aim was to evaluate the value of analysing spectral HR variation from ECG recordings obtained during autonomic cardiac measures and further the dynamic reactivity of the cardiac regulation in TLE patients during these tests as a tool to assess cardioregulatory function in these subjects.

## 2. Materials and Methods

### 2.1. Patients and Control Subjects

The study was carried out at the Outpatient Department of Neurology in the University Hospital, with the approval of the Ethics Committee of the local Medical Faculty. 

Thirty-six patients with TLE who were followed up in the Outpatient Clinic participated in the study after giving their informed consent. Eighteen patients suffered from recurrent TLE seizures despite adequate AED treatment, and 18 patients had well-controlled TLE (seizure-free on AEDs or <2 seizures/year). Both patient groups consisted of four male and 14 female consecutive patients. The control group consisted of 36 healthy age- and sex-matched subjects who were either hospital staff or were recruited by the staff. The subjects were eligible for the study if they did not have a disease or medication known to affect the ANS in their medical history and if the complete physical examination findings were normal. 

The results of heart rate and blood pressure (BP) responses to normal and deep breathing, tilt-table test, and isometric work conducted with present patients and control subjects were analysed and published previously [8]. In short, HR variation was lower in refractory TLE patients than in control subjects during normal breathing (*P* = 0.006) and tilting (*P* = 0.043), and HR was also low in well-controlled TLE patients compared to control subjects during tilting (*P* = 0.036). BP responses showed no differences between the patients and the control subjects. We were able to successfully perform the spectral analysis of HR variation from the ECG data of 36 patients, who represent a subset of the original 38 patients who participated in the autonomic cardiac function tests. The demographics of the study patients and the control subjects are shown in Table 1. 

Nine patients were taking CBZ, 11 patients were taking oxcarbazepine (OXC), and one patient was taking phenytoin, valproate, or lamotrigine each as monotherapy, and thirteen patients were on polytherapy as a combination of two to four of the above-mentioned antiepileptic drugs (AEDs). In addition, vigabatrin, clonazepam, and clobazam were all taken as part of polytherapy by one patient each. All thirteen patients who were on polytherapy had refractory TLE. 

All patients were carefully interviewed and clinically examined. The epilepsy type was classified according to the recommendations of the International League Against Epilepsy [37]. None of the patients showed any symptoms or signs of illness other than epilepsy known to affect the ANS. In general, the patients had neither excessive body weight, nor elevated blood pressure. Laboratory screening (liver and renal function, serum electrolytes, and basic hematologic parameters) was normal in all study patients. 

Patients abusing alcohol or drugs or taking any medication other than AEDs known to affect the ANS were excluded. Female patients who were pregnant or lactating were also excluded.

### 2.2. Methods

#### 2.2.1. Autonomic Cardiac Function Tests

All patients and control subjects underwent autonomic cardiac function testing that is based on HR and BP responses at rest and after various stimuli. All tests were performed under standardized conditions. The measurements were performed in a silent room with a temperature from 20 to 23°C between 9 a.m. and 12 a.m. The study subjects were asked not to smoke or drink coffee within two hours and not to use alcohol within 48 hours prior to the study. The interval between the various tests was standardized so that the next test did not start until the HR and BP had returned to the baseline level after the previous test. HR and BP responses at rest and after stimulation were recorded under the following conditions: normal breathing, deep breathing, the Valsalva maneuver, tilting, and isometric work (handgrip). ECG was first recorded during the normal breathing. Consecutive RR intervals were measured from the ECG for a period of 10 min and the standard deviation of the intervals was used as the test variable. In the tilt table test, the HR responses to quick passive tilting (2 s, 90°) and after tilting (7 min) were recorded. A more detailed description of the methodology of the autonomic cardiac test pattern of the patients and control subjects has been previously published [8, 35]. 

#### 2.2.2. HRV Analysis

Spectral analysis of the RR series of all patients and controls was performed with direct Fourier transformation [25]. The length of the time series was 10 min for the normal breathing test and 7 min for the tilt table test. RR intervals at rest and while standing were converted to a smoothed instantaneous RR time series at 4 Hz after manual editing of ectopic beats, and exact Hamming windowing (cos2) was applied before Fourier transformation. Total power (TP; 0.0033–0.40 Hz) and the components of high frequency (HF; 0.15–0.40 Hz) and low frequency (LF; 0.04–0.15 Hz) were identified for each subject visually from the Fourier series, and the HF band was manually selected according to the calculated actual respiration frequency spectrum. The LF : HF ratio was also calculated. The very-low-frequency band (VLF; 0.0033–0.04 Hz) was not included in the analysis because the adequate calculation of VLF requires a minimum of 12–16 hours of HR data [25].

#### 2.2.3. Statistics

**Figure 1:**
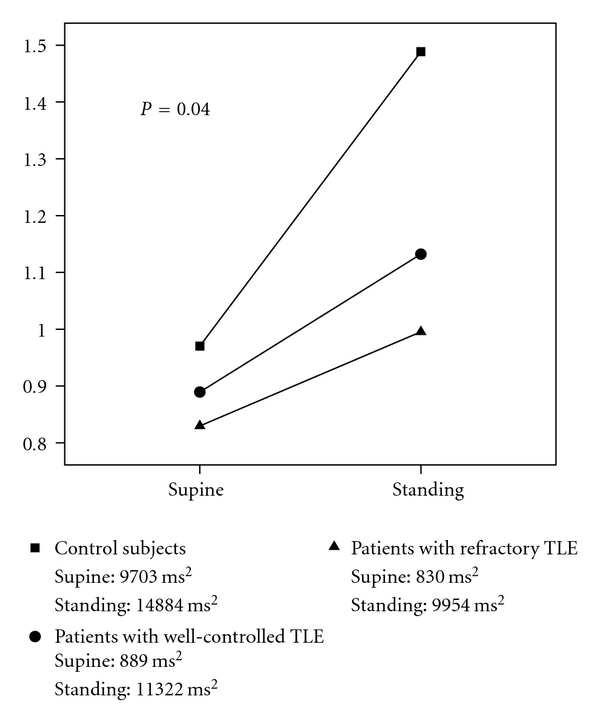
The change of total power from supine to standing in patients with refractory temporal lobe epilepsy (triangles), in patients with well-controlled temporal lobe epilepsy (circles), and the control subjects (squares). The values are estimated marginal means. The *P-*value describes the significance of the difference of the change from supine to standing position between the patient groups and the control subjects.

The nonparametric Mann-Whitney test was used in analysing unevenly distributed data. The difference of the changes of various measures of HR variation from supine to standing position was studied using the repeated measures analysis of variance. Because of unevenly distributed data, Tables 2 and 3 show the data as median (percentiles). However, as the data of analysis of repeated measures follows normal (Gaussian) distribution, the data in Table 4 is shown as mean (SD). The *P*-value of the interaction describing this change is reported in Table 4 and Figure 1. The *P*-value of ≤0.05 was considered to indicate a statistically significant difference. The statistical tests were performed with SPSS 14.0 software (SPSS Inc., Chicago, IL, USA).

## 3. Results

### 3.1. Supine Position

HF was decreased (*P* = 0.03) and the LF : HF ratio was increased (*P* = 0.003) in patients with TLE compared to the control subjects at rest. The results of various analyses at rest are shown in Table 2. Patients with refractory TLE had lower values of TP, HF, and LF and higher LF : HF ratio than patients with well-controlled epilepsy, but the differences were not statistically significant.

### 3.2. Standing Position

During the head-up tilting TP (*P* < 0.001), HF (*P* = 0.004) and LF (*P* = 0.001) were low in patients with TLE compared to the control subjects, but the LF : HF ratio did not differ between the patients and the control subjects. The results of various analyses of head-up tilt are shown in Table 3. Patients with refractory TLE presented lower values of TP, HF, and LF and higher LF : HF ratio than patients with well-controlled TLE. However, the differences were not statistically significant.

### 3.3. Change from Supine to Standing

The changes in the powers of different spectra during this test are shown in Table 4. The differences of TP in changing from supine to standing position in both patient groups and control subjects are also shown in Figure 1. Changing from supine to standing position resulted in significantly lower response in HR variation in patients with TLE compared to the controls in TP (*P* = 0.04) and LF (*P* = 0.006). The changes in HF and LF : HF ratio were similar in the TLE patients and the control subjects. Although the absolute values of changes in different spectra were systematically lower in patients with refractory TLE compared to patients with well-controlled TLE both in supine and tilting position, none of these differences were statistically significant (Table 4, Figure 1).

## 4. Discussion

This study was conducted to evaluate whether spectral analysis of HR variation from short ECG recordings obtained during autonomic cardiac function test pattern adds value to the assessment of the ANS in patients with TLE. The results suggest that this method may be a useful tool to detect alterations of autonomic cardiac functions. Patients with TLE had altered HR variation already in the resting state, and during the dynamic state (head-up tilt) these alterations were even more pronounced. Dynamic malfunction of the ANS of the TLE patients upon changing from supine to tilting position was easily detected by using spectral analysis of HR variation. At rest, the emphasis of malfunction was on vagal modulation on the HR whereas during tilting both the sympathetic and vagal modulation of the ANS in TLE patients showed inferiority to that of the control subjects. Interestingly, change from supine to standing position revealed mainly a breakdown of the sympathovagal balance in these patients. Although no statistically significant differences were detected between the two patient groups, there was a consistent trend towards lower values of the test parameters in the refractory TLE group, indicating a more disrupted cardioregulatory function compared to well-controlled TLE patients.

 Previous studies have shown altered cardioregulatory function in patients with TLE in cardiovascular function tests [5, 6, 8, 16, 38] and in spectral analyses of HR variation from 24-hour ECG recordings [4, 7, 10, 11, 13, 19]. Dysregulation of the cardiac autonomic functions has been described in association with various other diseases as well [20–24], and the prognostic significance of these findings has been established in heart and cerebrovascular diseases [20, 21, 24]. The present study agrees well with the previous studies in that TLE affects autonomic cardiac regulation. However, this study also shows that in addition to having an overall suppressing effect on the ANS function, especially refractory TLE seems to be associated with decreased reactivity of the cardiovascular ANS system. 

 The possible contribution of AEDs to ANS dysfunction in patients with epilepsy has not been clearly established. Previous work has shown that CBZ alters autonomic functions in epilepsy patients [39, 40]. In one study, slow withdrawal of AEDs in seizure-free epilepsy patients resulted in an increase of both parasympathetic and sympathetic function of HR variation [41]. Since in the current study most patients were taking CBZ or OXC, and refractory TLE patients were on polytherapy, it was not possible to analyse the effects of different AEDs on HR variability. However, it is possible that AEDs contributed to the detected alterations in the cardioregulation, and studies designed to analyse the effects of different AED regimens on autonomic cardiac function are needed. 

 The frequency domain measure is one of the linear analyses of the HR variation, where the power spectrum of RR intervals reflects the amplitude of the HR fluctuations at different oscillation frequencies [25]. The HF represents the vagal modulation of the heart, the LF band represents the sympathetic cardiac innervation and parasympathetic activity whereas both the VLF band and the LH : HF ratio are assumed to represent the sympathovagal balance [25]. The frequency domain analysis of HR variation, along with various other indices are typically analysed from 24-hour ambulatory ECG-recordings. Although this method has the advantage that the information concerning the VLF band is gathered over a longer time period [25], the ambulatory recording may be interfered by stationary irregularities and artefacts in consequence of the patients carrying the device during their daily activities [42]. On the other hand, analysis of HR variation from ECGs obtained during the provocative autonomic testing, as shown in this study, may provide valuable information on the reactivity of the autonomic cardioregulation, and this method only requires a short period of time under standardised conditions.

 It is well known that various cortical and subcortical autonomic areas control the cardiovascular function [18, 29, 43–46]. Chronic epileptiform discharges or epileptic foci in these areas may produce potentially life-threatening cardiac arrhythmias both ictally and interictally as cardiovascular regulation is, in fact, a function of neuronal activity in the cerebral cortex, the amygdale, and the medullary reticular formation [18, 29, 43–46]. It has been suggested in the context of increased preictal HR that in patients with primarily more instability and activation in cortical networks surrounding the seizure onset zone will simultaneously increase sympathetic tone and risk for generalization of the seizure, since noradrenergic function may be needed for the anticonvulsive effect of the vagus nerve stimulation, at least in animals [47]. However, on the other hand, it has also been shown that secondarily generalized seizures lead to higher ictal HR and sustained postictal tachycardia with enhanced shortening of QT-interval possible showing that cardiac alterations are in fact a link between generalized seizures and SUDEP [48]. In this study, TLE patients showed altered sympathovagal balance in the interictal state. During the dynamic test situation, especially refractory TLE patients showed decreased reactivity of the cardiac autonomic functions with the breakdown of sympathovagal balance. In the future it would be interesting to study interictal and ictal cardiac measures to evaluate whether interictal changes in HR correlate with ictal HR changes in the same patient. 

 Chronic epilepsy with recurrent generalized seizures is known to be associated with a high risk of SUDEP [49, 50]. SUDEP is the utmost consequence of numerous different factors that precipitate fatal complications of an epileptic activity in a predisposed person [27, 28, 30, 31, 49–51]. As the knowledge and understanding of the pathophysiological background of SUDEP increases, it is necessary to continue to develop methods to study ANS function in patients with epilepsy along with identifying the clinical risk markers. These methods may eventually provide tools to identify epilepsy patients with ANS dysfunction and risk of SUDEP and allow preventive actions.

 The limitation of our study was the small number of patients that were evaluated. It is possible that a higher number of patients would have established more statistically significant differences between the study groups. Because of the unbalanced male-to-female ratio we cannot conclude that these results apply to both genders. 

 In conclusion, spectral HR variation during autonomic function tests is a useful tool to study autonomic cardiac regulation in patients with epilepsy. When the autonomic cardiac function tests are performed under standardized laboratory conditions according to a regular protocol, the method is reliable. The reactivity of the cardioregulatory functions can be assessed more carefully by including spectral analysis of HR variation into the study protocol. TLE patients showed a sympathovagal malfunction during the dynamic test situation. This was even more pronounced in patients with refractory TLE. Although reduced HR variation is not considered a direct indication of elevated risk of SUDEP, the inferior capacity of the ANS to react during dynamic situations may be a manifestation of autonomic imbalance during generalized epileptic seizures.

##  Conflict of Interests

The authors declared that there are no conflict of interests to disclose.

## Figures and Tables

**Table 1:** Demographics of the study patients and the control subjects.

	No. of patients/control subjects	Age, years, mean (SD)	Duration of epilepsy years, mean (SD)
Patients with refractory TLE	18	33 (7)	22 (11)

Patients with well-controlled TLE	18	35 (6)	15 (10)

Patients, total	36	34 (7)	19 (11)

Control subjects	36	34 (7)	

**Table 2:** Heart rate variation at rest in patients with temporal lobe epilepsy (TLE) and the control subjects. The *P*-value presents the differences between all patients and the control subjects.

Measure/ variable (ms^2^)	Patients with refractory TLE	Patients with well-controlled TLE	All patients	Control subjects	*P*-value (Mann-Whitney)
TP	7417 (6276, 11043)	8093 (7016, 1044)	7991 (6292, 10521)	9518 (6742, 13923 )	0.33
HF	2471 (1752, 3622)	2954 (2092, 4397)	2729 (1979, 4265)	3954 (2855, 5705)	0.03
LF	2284 (1864, 3505)	2855 (2117, 3449)	2691 (1876, 3416)	2604 (1583, 4091)	0.54
LF : HF ratio	0.90 (0.76, 1.25 )	0.91 (0.70, 1.12)	0.90 (0.75, 1.12)	0.84 (0.65, 1.16)	0.003

Values are median (25., 75. percentile); TP: total power; HF: high frequency; LF: low frequency.

**Table 3:** Heart rate variation during head-up tilt in patients with temporal lobe epilepsy (TLE) and the control subjects. The *P*-value presents the differences between all patients and the control subjects.

Measure/ variable (ms^2^)	Patients with refractory TLE	Patients with well-controlled TLE	All patients	Control subjects	*P*-value (Mann-Whitney)
TP	8897 (7076, 1444)	10822 (8543, 12619)	10314 (8182, 13359)	14022 (11262, 17916)	<0.001
HF	2175 (1250, 3918)	2428 (2017, 3113)	2347 (1810, 3336)	3221 (2482, 4780)	0.004
LF	4248 (2525, 6035)	4326 (3682, 5687)	4248 (3198, 5633)	5800 (4308, 7142)	0.001
LF : HF ratio	1.73 (1.26, 2.31)	1.73 (1.41, 2.47)	1.73 (1.36, 2.33)	1.64 (1.35, 2.34)	0.85

Values are median (25., 75. percentile); TP: total power; HF: high frequency; LF: low frequency.

**Table 4:** The change in heart rate variation from supine to standing position in patients with temporal lobe epilepsy (TLE) and the control subjects. The *P*-value presents the differences between all patients and the control subjects.

Measure/ variable (ms^2^)	Patients with refractory TLE	Patients with well-controlled TLE	All patients	Control subjects	*P*-value (interaction)
TP	173 (438)	243 (222)	208 (344)	504 (596)	0.01
HF	−46 (175)	−70 (122)	−58 (149)	−68 (282)	0.85
LF	157 (231)	169 (142)	163 (189)	351 (279)	0.001
LF : HF ratio	0.90 (0.95)	0.89 (0.83)	0.89 (0.88)	1.1 (0.70	0.23

Values are mean ± SD; TP: total power; HF: high frequency; LF: low frequency.
